# Supplementary material for: Organomegalies as a predictive indicator of leukemia cutis in patients with acute myeloid leukemia
Source: PLoS One. 2024 Feb 16;19(2):e0297805. doi: 10.1371/journal.pone.0297805 (PMC10871476; doi:10.1371/journal.pone.0297805)
Supplement: S1 Table — (DOCX) [file pone.0297805.s001.docx]

**S1 Table.** Balance diagnostics for case-control matching by propensity score

| **Characteristic** | **Before matching** | | | **After matching** | | |
| --- | --- | --- | --- | --- | --- | --- |
|  | **AML with LC** | **AML without LC** | **SMD** | **AML with LC** | **AML without LC** | **SMD** |
|  | **Overall survival propensity score matched analysis** | | | | | |
|  | **(n=31)** | **(n=69)** |  | **(n=26)** | **(n=52)** |  |
| Age ± SD | 48.5 **±** 12.8 | 46.4 **±** 13.5 | 0.16 | 47.3 **±** 12.5 | 46.6 **±** 13.1 | 0.05 |
| Male | 14 (45.2) | 33 (47.8) | - 0.05 | 13 (50.0) | 26 (50.0) | - 0.05 |
| Cytogenetic risk |  |  |  |  |  |  |
| Favorable | 4 (12.9) | 9 (13.0) | - 0.004 | 3 (11.5) | 5 (9.6) | 0.06 |
| Intermediate | 21 (67.7) | 49 (71.0) | - 0.07 | 19 (73.1) | 40 (76.9) | - 0.07 |
| Adverse | 6 (19.4) | 11 (15.9) | 0.09 | 4 (15.4) | 7 (13.5) | 0.03 |
|  | **Relapse-free survival propensity score matched analysis** | | | | | |
|  | **(n=24)** | **(n=55)** |  | **(n=24)** | **(n=46)** |  |
| Age ± SD | 49.7 ± 12.0 | 46.6 ± 13.4 | 0.26 | 49.7 ± 12.0 | 48.3 ± 12.9 | 0.07 |
| Male | 13 (54.2) | 26 (47.3) | 0.13 | 13 (54.2) | 23 (50.0) | 0.04 |
| Cytogenetic risk |  |  |  |  |  |  |
| Favorable | 4 (16.7) | 8 (14.6) | 0.06 | 4 (16.7) | 8 (17.4) | 0.00 |
| Intermediate | 16 (66.7) | 41 (74.6) | - 0.17 | 16 (66.7) | 32 (69.6) | 0.00 |
| Adverse | 4 (16.7) | 6 (10.9) | 0.15 | 4 (16.7) | 6 (13.0) | 0.00 |

**Notes:-** Data are presented as n (%) unless stated otherwise.

**Abbreviations:** *AML* acute myeloid leukemia, *LC* leukemia cutis, *SMD* standardized mean difference.
