# Supplementary material for: Organomegalies as a predictive indicator of leukemia cutis in patients with acute myeloid leukemia
Source: PLoS One. 2024 Feb 16;19(2):e0297805. doi: 10.1371/journal.pone.0297805 (PMC10871476; doi:10.1371/journal.pone.0297805)

**S2 Fig.** Propensity score-adjusted Kaplan–Meier survival curve illustrating relapse-free survival comparing leukemia cutis to non-leukemia cutis AML patients receiving intensive chemotherapy


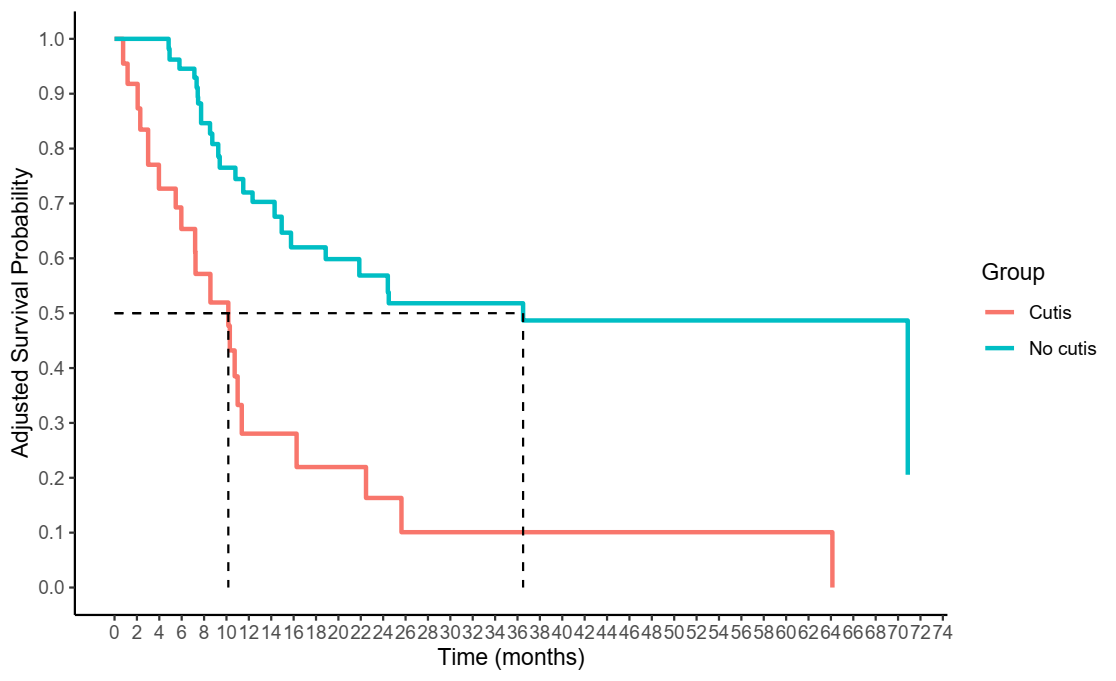

Supplement: S2 Fig — (DOCX) [file pone.0297805.s003.docx]
